# Sex‐dependent role of 20‐HETE synthesis in outcome from ischemic stroke in rats

**DOI:** 10.14814/phy2.70762

**Published:** 2026-02-03

**Authors:** Rongrong Zhang, Yanrong Shi, Suyi Cao, Raymond C. Koehler, Zeng‐Jin Yang

**Affiliations:** ^1^ Department of Anesthesiology and Critical Care Medicine Johns Hopkins University Baltimore Maryland USA; ^2^ Department of Neurology The First Affiliated Hospital of Chongqing Medical University Chongqing China

**Keywords:** castration, cytochrome P450 4A, middle cerebral artery occlusion, ovariectomy, oxygen–glucose deprivation, ω‐hydroxylase

## Abstract

The lipid mediator 20‐HETE is produced by ω‐hydroxylation of arachidonic acid mediated by cytochrome P450 (CYP) enzymes including CYP4A, which has four distinct isoforms in rodents. Several laboratories demonstrated that 20‐HETE synthesis inhibition reduces infarct volume following middle cerebral artery occlusion (MCAO) in male animals. Here, we investigated whether neuroprotection with the 20‐HETE synthesis inhibitor HET0016 administered after transient MCAO in rats differs by sex and whether ischemia differentially induces *Cyp4a* genes in a sex‐dependent manner. HET0016 significantly improved sensorimotor performance and reduced infarct volume compared to vehicle treatment in males. However, these improvements were less consistent in females. *Cyp4a2* and *Cyp4a3* genes were detected at similar levels in brain tissue from male and female rats undergoing sham surgery or MCAO/reperfusion. Interestingly, the *Cyp4a8* gene was detectable in intact and castrated males and increased 3‐4‐fold after MCAO. In contrast, *Cyp4a8* was undetectable in brains of intact or ovariectomized female rats. Oxygen–glucose deprivation in cultured murine neurons revealed male‐selective induction of the homolog gene *Cyp4a12a*, the knockdown of which blocked the increase in 20‐HETE. These results indicate that innate male‐selective *Cyp4a* gene induction and 20‐HETE signaling are significant factors that can contribute to sex differences in the outcomes from ischemic stroke.

## 1 INTRODUCTION

Epidemiological studies reveal sex differences in stroke (Bushnell et al., 2018). Outcomes from stroke are reported to be worse in young males than young females, but this shifts in the aging population where women have a worse outcome (Roy‐O'Reilly & McCullough, 2018). The preclinical literature also indicates sex differences in outcomes from ischemic stroke (Chauhan et al., 2017). Studies of transient middle cerebral artery occlusion (MCAO) in young mice and rats often report smaller infarcts in females compared to males (Alkayed et al., 1998; McCullough et al., 2005). Estrogen plays an important role in this sex difference because ovariectomy increases infarct volume, and estrogen replacement in ovariectomized rats can mitigate this worsening of infarct volume. However, genetic factors may also play a role in inherent sex differences. Inherent sex differences are evident in primary neuronal cultures exposed to excitotoxic stimulation or oxygen–glucose deprivation (OGD), with male neurons exhibiting greater oxidative and nitrosative stress and cell death (Du et al., 2004; Fairbanks et al., 2012). Likewise, in organotypic hippocampal slice cultures exposed to OGD, neuronal death was greater in those derived from male rats than from female rats, which was also attributable to nitrosative stress (Li et al., 2005). Thus, some of the sex differences are not attributable to differences in perfusion and circulating immune cells.

A body of work from several laboratories has implicated 20‐hydroxyeicosatetraenoic acid (20‐HETE) as a factor contributing to cerebral ischemic injury. These include studies using 20‐HETE synthesis inhibitors, such as HET0016 (Seki et al., 2005) and TS‐011, in models of transient MCAO in rats (Miyata et al., 2005; Omura et al., 2006; Poloyac et al., 2006; Renic et al., 2009), in monkeys with an embolic clot injection (Omura et al., 2006), and in studies on piglets subjected to hypoxia‐ischemia (Yang et al., 2012; Zhu et al., 2015). The brain concentration of 20‐HETE increases after transient MCAO (Renic et al., 2009; Tanaka et al., 2007), and the increase is blocked by HET0016 administration (Poloyac et al., 2006). However, these studies used male animals and did not report infarct volume or neurobehavior outcome specifically in female animals. Thus, it is unknown whether inhibition of 20‐HETE synthesis is protective in females with stroke.

20‐HETE is formed by the ω‐hydroxylation of arachidonic acid by isoforms in the CYP4A family. In the rat, *Cyp4a1*, *a2*, *a3*, and *a8* genes have been identified; the genes in mice are *Cyp4a10*, *4a12a*, *4a12b*, and *4a14* (Singh et al., 2007; Stec et al., 2003). 20‐HETE has been primarily studied in renal and vascular smooth muscle function. In arteries, 20‐HETE acts through GPR75 (Garcia et al., 2017) to promote PKC‐dependent activation of L‐type Ca^+2^ channels, depolarization, and constriction (Roman, 2002). Sex differences in vascular regulation of CYP4A isoforms that synthesize 20‐HETE from arachidonic acid have been found in the renal vasculature and along the nephron (Muller et al., 2007; Stec et al., 2003). Androgen‐induced hypertension and endothelial dysfunction are associated with *Cyp4a8* upregulation in renal arteries in rats (Nakagawa et al., 2003; Singh et al., 2007) and with its homolog *Cyp4a12* in mice (Holla et al., 2001). Whether similar sex‐dependent effects on the induction of CYP4A isoforms exist in brain has not been investigated.

The presence of CYP4A protein in neurons was demonstrated by immunohistochemistry in cultured mouse neurons (Zhang et al., 2018; Zhang, Falck, et al., 2017), in rat organotypic hippocampal slice cultures (Renic et al., 2012), and in rat and piglet brain (Gonzalez‐Fernandez et al., 2020; Yang et al., 2012). OGD increased the level of 20‐HETE, reactive oxygen species (ROS) formation, and neuronal cell death in the hippocampal slice culture. All of these effects were reduced by HET0016 application and replicated by applying a stable 20‐HETE mimetic without OGD. However, CYP4A antibodies do not distinguish the individual isoforms. To determine the specific *Cyp4a* genes expressed in neurons and to better isolate the effect of OGD on neurons, our group performed OGD in mouse primary neuronal cultures (Zhang, Falck, et al., 2017). Gene expression of *Cyp4a10* and *Cyp4a12a* was present in control neurons and increased within 1 h of reoxygenation after 1‐h OGD. The other mouse isoforms *Cyp4a12b* and *Cyp4a14* were not detectable in neurons but were noticeable in renal microsomes. As in the rat hippocampal slice cultures, HET0016 reduced ROS formation and neuronal cell death after OGD in primary neuronal culture. Applying a 20‐HETE stable mimetic in the presence of HET0016 and OGD restored ROS formation and increased neuronal cell death, consistent with HET0016 acting as an inhibitor of 20‐HETE synthesis rather than as an antagonist. Because the studies in primary neuronal culture were of mixed sex, the results did not differentiate potential sex differences.

The objectives of the current study were (1) to determine whether there is a sex‐dependent effect of CYP4A inhibition with HET0016 on infarct volume and neurobehavior outcome, (2) to determine the changes in gene expression of CYP4A isoforms in vivo in rat brains after MCAO and whether CYP4A isoform expression is influenced by sex and androgen status, and (3) to extend the previous work on OGD in mixed‐sex mouse neuronal cultures and determine whether OGD upregulates *Cyp4a* genes differentially in sex‐stratified neuronal cultures. We also investigated whether siRNA‐induced knockdown of the upregulated *Cyp4a12a* gene reduces 20‐HETE generation after OGD and whether the observed upregulation of *Cyp4a* genes after OGD is specific for neurons or also occurs in cultured astrocytes and microglia.

## 2 METHODS

### 2.1 General animal care procedures

All procedures on animals were approved by the Johns Hopkins Animal Care and Use Committee and conformed to the NIH guidelines for use of animals in research. Protocols adhered to the ARRIVE guidelines. In vivo experiments were performed on Sprague Dawley rats (Charles River Laboratories) at approximately 3 months of age. The rats were housed in pairs and maintained in the animal facility of Johns Hopkins University School of Medicine in ventilated racks with a 12‐h light cycle (with procedures performed during the light phase) in a temperature (20°C–25°C) and humidity (30%–70%) controlled room. They had ad libitum access to water and standard chow (5v75—PicoLab Verified 75 IF, Lab Diet). All surgeries were performed using aseptic techniques under general anesthesia with isoflurane in oxygen‐enriched air and spontaneous respiration. Incisions sites were infiltrated with bupivacaine. Rectal temperature was maintained at 37°C with a heating pad. Postoperative analgesia was provided with buprenorphine. The target sample size was set at 8 for 3‐day survivors for measurements based on the coefficient of variation of infarct volume in previous work (Renic et al., 2009) and an effect size of 20% of hemisphere volume. For tissue gene expression measurements, a sample size of 4 was assumed to be sufficient for detecting physiologically meaningful changes of at least 50%.

### 2.2 MCAO model

The MCAO procedure has been previously used in our lab (Cao et al., 2009, 2017; Klaus et al., 2010; Li et al., 2011; Zhang et al., 2012; Zhang, Li, et al., 2017). A midline incision was made in the neck, and the right common carotid artery was exposed by blunt dissection. The right external carotid artery was dissected and doubly ligated with 4–0 silk suture. The occipital artery branch of the external carotid artery was also isolated and ligated. The external carotid artery was cut between the ligatures. A 4–0 monofilament nylon suture with the tip enlarged with silicone glue to 0.43 mm diameter over a 3–4 mm length (Doccol Corp., Sharon, MA) was inserted into the stump and advanced approximately 18 mm into the internal carotid artery until resistance was felt within the Circle of Willis. The filament was secured in place with a 4–0 silk suture, the neck incision was closed, anesthesia was discontinued and the rat was returned to a cage with bedding material. Prior to the end of the 120‐min occlusion period, the rat was re‐anesthetized, the filament was withdrawn, 2 mL of lactated Ringer's solution was injected subcutaneously, and the rat was returned to its home cage with its cagemate. Rats undergoing sham surgery underwent the same anesthesia and surgery procedures except that the filament was not advanced into the internal carotid artery. Two sets of in vivo experiments were performed: one in which neurobehaviour and infarct volume were assessed, and another in which *Cyp4a* gene expression in brain was assessed with RT‐PCR.

### 2.3 Castration

Some male rats underwent castration 2 weeks prior to sham surgery or MCAO surgery. To remove the testes, a 1‐cm skin incision was made in the midline of the scrotum along the septum, and the skin of the scrotum was detached by blunt dissection (Toung et al., 1998). On each side, an incision was made through the sac in which each testis was enclosed, and the connective tissue leading to the testis, including the fat pad, testicular vein, and vas deferens, was doubly ligated. The testis was cut away from the connecting tissue and the ligated connective tissue was returned to the body cavity.

### 2.4 Slow‐release pellet implantation

For castrated rats used for RT‐PCR measurements after sham or MCAO surgery, some received androgen replacement to specifically evaluate the role of testosterone on *Cyp4a* expression in the brain. After completing the castration procedure, the rat was turned to the prone position and a 1‐cm incision was made in the skin. A slow releasing pellet containing either 5α‐dihydrotestosterone (DHT, 50 mg, 21‐day release; Cat# A‐161, Innovative Research of America, Sarasota, FL) or placebo (Cat# C‐111, Innovative Research of America) was implanted subcutaneously. DHT rather than testosterone was used because it is not transformed by aromatase into estrogen (Cheng et al., 2007).

### 2.5 Ovariectomy

For female rats used for RT‐PCR measurements, some underwent ovariectomy to determine if there was a sex hormone effect on *Cyp4a* gene expression after stroke. To remove the ovaries, a 1‐cm skin incision was made in the area that bisects the angle formed by the ribs and spinal column (Alkayed et al., 1998; Toung et al., 1998). A blunt dissection was made into the muscle layer, and a small incision was made in the peritoneum. The ovary, its associated fat pad, and the uterine horn to which it is attached were pulled out of the opening in the muscle and ligated. The ovary and most of the fat pad were cut away. The ligated uterine horn was returned to the body cavity and the incision of the peritoneum and the skin was closed with suture. Because the existing literature does not support a role of female sex hormones on *Cyp4a* gene expression, we did not pursue estrogen replacement in this experiment.

### 2.6 Protocol for neurobehavior and infarct volume measurements

This experiment was designed to assess sex‐dependent and androgen‐dependent effects of CYP4A inhibition with HET0016 on infarct volume and neurobehavior outcome. An ovariectomy group was not included in this experiment because none of the *Cyp4a* genes were found to be upregulated in intact female rats or cultured female neurons. HET0016 [N‐hydroxy‐N‐4‐butyl‐2‐methylphenylformamidine] (Cat# 75780, Cayman, Ann Arbor, MI) was dissolved in 45% (2‐hydroxypropyl)‐β‐cyclodextrin (H5784, Millipore Sigma, St. Louis, MO) in saline at 37°C. HET0016 dissolved in this solvent at a dose of 1 mg/kg decreases 20‐HETE concentration in rat brain (Mu et al., 2008). The MCAO rats were randomized to receive 1 mg/kg HET0016 or vehicle intraperitoneally at 10 min prior to reperfusion and a second dose at 3 h of reperfusion. This dosing regimen has been used previously (Renic et al., 2009). Because 20‐HETE availability is oxygen‐dependent (Gebremedhin et al., 2008; Harder et al., 1996), 20‐HETE signaling may be a component of reperfusion injury. Thus, we elected to start treatment with HET0016 at reperfusion from MCAO. Intact male, castrated male, and intact female rats underwent sham surgery or 2‐h MCAO. Over the 3‐day recovery period, neurological deficits were scored wherein 0 = normal, 1 = turning the body to one side when holding the rat by the tail, 2 = circling behavior, 3 = inability to bear weight on one side, and 4 = no spontaneous movement. Turning preference on the corner test was performed during the week before sham or MCAO surgery and again 3 days after surgery by an observer blinded to treatment.

For the corner test, the rat was placed at the entrance of two opaque boards, each with a dimension of 45 × 25 cm and constructed to form a corner of 30°. As the rat moves into the corner, the whiskers on both sides are brushed along the walls concurrently. The rat usually rears forward and upward and turns to face the open end. The turns involving full rearing to the left or right were counted over 10 trials. The corner test turning preference index was calculated as the fraction of turns to the right.

Rats that underwent MCAO also had infarct measurements on brains harvested at 3 days. Fresh coronal sections (2 mm thick) were stained with triphenyltetrazolium chloride (TTC, T8877, Millipore Sigma). Using Image J software, the area of pallor on each section and the area of each hemisphere was measured on each section to obtain infarct volume, which was corrected for swelling (Belayev et al., 2003).

### 2.7 Protocol for CYP4A gene expression measurements

This experiment was designed to assess sex‐dependent effects of MCAO and reperfusion on gene expression of the CYP4A isoforms. Groups included males, castrated males, castrated males treated with DHT to replace testosterone, females, and ovariectomized females. These groups were further divided into those undergoing sham surgery or 2 h MCAO and 6 h of reperfusion. MCAO or sham surgery was performed 10–14 days after castration and ovariectomy, which allows sufficient time for sex hormones to decline (Alkayed et al., 2001; Toung et al., 1998) and for DHT to alter ischemic gene expression (Cheng et al., 2007). After 6 h of reperfusion or an equivalent time in sham cohorts, the rats were anesthetized and underwent transcardial perfusion with phosphate‐buffered saline. The ipsilateral cerebral hemisphere was processed for the quantitative RT‐PCR analysis of the rat *Cyp4a* isoform genes (*4a1*, *4a2*, *4a3*, and *4a8*). The 6‐h reperfusion time point represents a time when injury is spreading into the penumbral region. In mouse primary neuronal culture, gene expression increases within 1 h and protein within 3–6 h of reoxygenation after OGD (Zhang, Falck, et al., 2017). The expression of *Cyp4a10* and *Cyp4a12a* increased by more than 3 SD; allowing for more variability in vivo, we expected a sample size of 4 to provide >80% power for detecting 50% increases relative to sham. Because CYP4A expression in kidney is influenced by androgens (Nakagawa et al., 2003; Singh et al., 2007), we did not expect to see an effect of ovariectomy on gene expression. Hence, we did not test the effect of estrogen supplementation in ovariectomized females.

### 2.8 Quantitative reverse transcriptase‐PCR (qRT‐PCR) on rat tissue

Total RNA was extracted from ipsilateral rat brain tissues using the RNeasy Plus Mini Kit (Cat# 74134, Qiagen, Germany). Complementary DNA (cDNA) was synthesized from 1 μg of total RNA using the High‐Capacity RNA‐to‐cDNA Kit (Cat# 4387406, ThermoFisher Scientific). Quantitative PCR (qPCR) was conducted in triplicate to measure total cDNA using the Power SYBR Green PCR Master Mix (Cat# 4367659, ThermoFisher Scientific) on the QuantStudio 3 real‐time PCR system (ThermoFisher Scientific) following standard protocols. Target gene expression was analyzed across three independent experiments. Relative expression levels of *Cyp4a1*, *Cyp4a2*, *Cyp4a*, and *Cyp4a8* mRNA were calculated using the quantification cycle (Cq) values normalized to glyceraldehyde‐3‐phosphate dehydrogenase (GAPDH) mRNA, employing the 2^−ΔΔCq^ method. The forward (F) and reverse (R) primer sequences used for amplification of the target genes were based on previous studies for rat *Cyp4a* (Dunn et al., 2008) and for rat GAPDH (Kanungo et al., 2008) and were synthesized by Integrated DNA Technologies, Inc. (Table 1). RNA from rat kidney tissue served as a positive control (Roman, 2002).

**TABLE 1:** CYP4A primers used for rat brain and mouse cell cultures.

Gene	Primer
*Rat*	
Cyp4a1 forward	5′‐CTC TTA CTT GCC AGA ATG GAG AA‐3′
Cyp4a1 reverse	5′‐GAC TTG GAT ACC CTT GGG TAA AG‐3′
Cyp4a2 forward	5′‐GTC CCC ATG CCA AGA CTT GT‐3′
Cyp4a2 reverse	5′‐GTC TGG AGT AAA AGC TTT GGA GCT‐3′
Cyp4a3 forward	5′‐CAG TGG CTC TCA GGG AGC AAA AC‐3′
Cyp4a3 reverse	5′‐GCA AGG AAT TGA TAA ATT CCA GAA GCC‐3′
Cyp4a8 forward	5′‐ATC CAG AGG TGT TTA CCC TTA T‐3′
Cyp4a8 reverse	5′‐AAT GAG ATG TGA GCA GAT GGA GT‐3′
GADPH forward	5′‐GAC ATG CCG CCT GGA GAA AC‐3′
GADPH reverse	5′‐AGC CCA GGA TGC CCT TTA GT‐3′
*Mouse*	
Cyp4a10 forward	5′‐TCC AGG TTT GCA CCA GAC TCT‐3′
Cyp4a10 reverse	5′‐TCC TGG CTC CTC CTG AGA AG‐3′
Cyp4a12a forward	5′‐GCC TTA TAC GGA AAT CAT GGC‐3′
Cyp4a12a reverse	5′‐TGG AAT CCT GGC CAA CAA TC‐3′
Cyp4a12b forward	5′‐CCT TCT ACG GAA ATC ATG GCA GA−3′
Cyp4a12b reverse	5′‐TGG AAT CCT GGC CAA CAA TC‐3′
Cyp4a14 forward	5′‐CAA GAC CCT CCA GCA TTT CC‐3′
Cyp4a14 reverse	5′‐GAG CTC CTT GTC CTT CAG ATG GT‐3′
GADPH forward	5′‐TGT GTC CGT CGT GGA TCT GA‐3′
GADPH reverse	5′‐CCT GCT TCA CCA CCT TCT TGA‐3′

### 2.9 Mouse brain cell culture and OGD

Primary cortical neurons were isolated from embryonic day 17 (E17) C57BL/6 mouse brains as previously described (Fairbanks et al., 2013; Zhang, Falck, et al., 2017). The sex of embryos was identified by the presence of striated testes (male) or uterus and fallopian tubes (female), and confirmed by PCR amplification of X‐linked Jarid1c (331 bp) and Y‐linked Jarid1d (302 bp) genes (Clapcote & Roder, 2005). Astrocytes and microglia were prepared from postnatal day 1 mouse brains according to established protocols (Bronstein et al., 2013; Schildge et al., 2013). Antibodies against cytochrome P450 4A and NeuN (Cat# ab3573 and ab134014, Abcam), GFAP (Cat# 3670, Cell Signaling Technology), and CD11b (Cat# NB600‐1327, Novus Biologicals) and secondary antibodies (Cat# A78948, A31572, A21202, and A21208, ThermoFisher Scientific) were used to identify CYP4A, neurons, astrocytes, and microglia, respectively.

For OGD, cells were incubated in glucose‐free DMEM and placed in a hypoxic chamber (95% N_2_/5% CO_2_) at 37°C for 1 h (neurons) or 3 h (astrocytes and microglia). These durations were chosen to produce partial cell death of approximately 40%–50% in the respective cultures. Reoxygenation was achieved by restoring cells to normal culture conditions with glucose and 95% air/5% CO_2_ for the indicated periods. Control cells were treated with equivalent medium exchanges without OGD exposure. In some experiments, neurons were transfected with Silencer Select Cyp4a12a siRNA or a Cy3‐labeled negative control (Cat# 4390771 and AM4621, Thermo Fisher Scientific) 3 days before OGD using an overnight transfection.

### 2.10 Quantitative reverse transcriptase‐PCR (qRT‐PCR) on murine cells

Total RNA was extracted using the RNeasy Plus Mini Kit (Cat# 74134, Qiagen, Germany). Complementary DNA (cDNA) was synthesized from 1 μg of total RNA using the High‐Capacity RNA‐to‐cDNA Kit (Cat# 4387406, Thermo Fisher Scientific). Quantitative PCR was performed in triplicate using Power SYBR Green PCR Master Mix (Cat# 4367659, Thermo Fisher Scientific) on a QuantStudio 3 Real‐Time PCR System following standard protocols. Relative mRNA expression of Cyp4a10, Cyp4a12a, Cyp4a12b, and Cyp4a14 was normalized to Gapdh using the 2^−ΔΔCq^ method. Primer sequences were based on published studies (Zhang & Klaassen, 2013) and were synthesized by Integrated DNA Technologies, Inc. (Table 1).

### 2.11 20‐HETE quantification

Cells were homogenized in buffer containing ~0.1 mM triphenylphosphine (TPP) to prevent oxidation. Homogenates were acidified to pH 3–4 with acetic acid and extracted three times with equal volumes of ethyl acetate. The combined organic phases were evaporated to dryness under nitrogen, and residues were reconstituted in 20 μL N,N‐dimethylformamide followed by 0.5 mL sample dilution buffer. Samples and serial standard dilutions were loaded onto ELISA plates to determine 20‐HETE concentrations according to the manufacturer's instructions (BioTarget 20‐HETE ELISA Kit Cat# 20H1, Detroit R&D, Detroit, MI).

### 2.12 Statistical analysis

For RT‐PCR measurements, sample sizes were small and the non‐parametric Mann–Whitney test was used for comparisons between MCAO cohorts and their respective sham groups. Because the discreet neurologic deficit scores and corner test turning preference index did not meet the criteria for normality and equal variance, the outcomes between the vehicle and HET0016 groups within each sex were analyzed with the Mann–Whitney test at the 0.05 significance level. The log rank Kaplan–Meier survival analysis was performed on the groups that received treatment after MCAO. For cell culture studies, comparisons were made between control cultures and cultures exposed to OGD with the Mann–Whitney test. Comparisons between male‐ and female‐derived cultures exposed to the same conditions were compared with the Mann–Whitney test.

## 3 RESULTS

### 3.1 Effect of inhibition of CYP4A inhibition on stroke outcome

**FIGURE 1:**
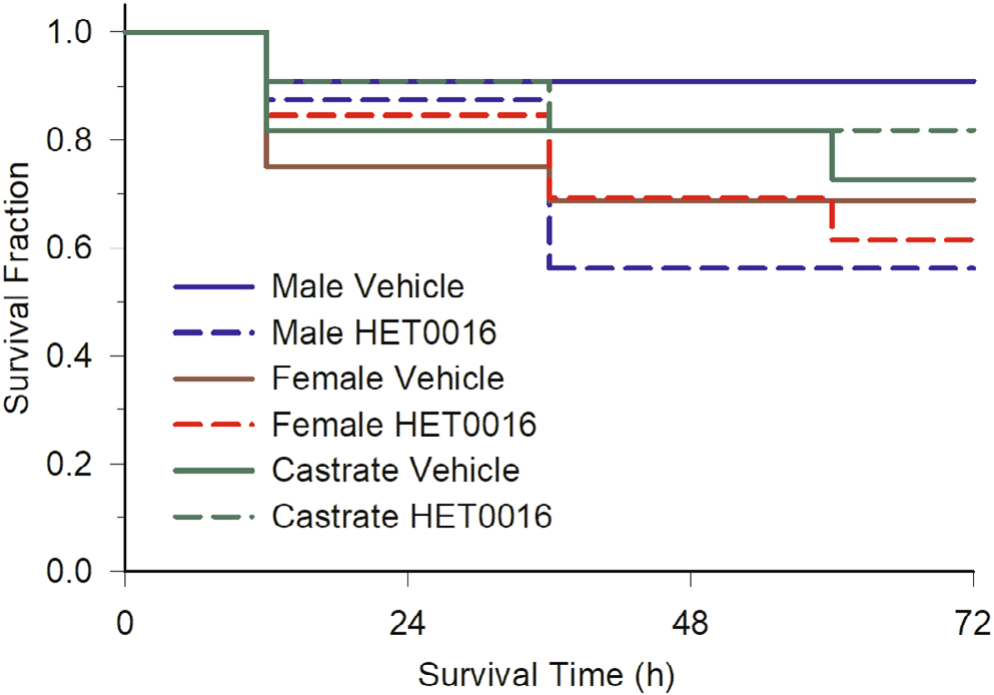
Fraction of rats that survived at 1, 2, and 3 days after middle cerebral artery occlusion and reperfusion and completing the vehicle or HET0016 treatment (1 mg/kg intraperitoneally at 10 min prior to reperfusion and a second dose at 3 h of reperfusion). Those rats completing treatment included 11 in the male vehicle group, 16 in the male HET0016 group, 16 in the female vehicle group, 13 in the female HET0016 group, 9 in the castrate vehicle group, and 12 in the castrate HET0016 group. The log‐rank Kaplan–Meier analysis indicated that survival was not different among the groups (*p* = 0.49).

To determine if there was a sex difference in the effect of inhibition of CYP4A ω‐hydoxylation by HET0016 treatment on outcomes from MCAO, male and female rats underwent sham surgery or MCAO surgery followed by treatment with vehicle or HET0016 at reperfusion and again at 3 h of reperfusion. Because expression of some Cyp4a isoforms is known to be positively affected by androgens, we also included cohorts of castrated males in this experiment. With vehicle or HET0016 treatment after MCAO, some mortality occurred, primarily over the first 2 days in those that completed treatment after MCAO (Figure 1). The log rank Kaplan–Meier analysis indicated no significant difference among the 4 MCAO groups (*p* = 0.49). Sham animals had no mortality after recovery and were not included in the analysis.

**FIGURE 2:**
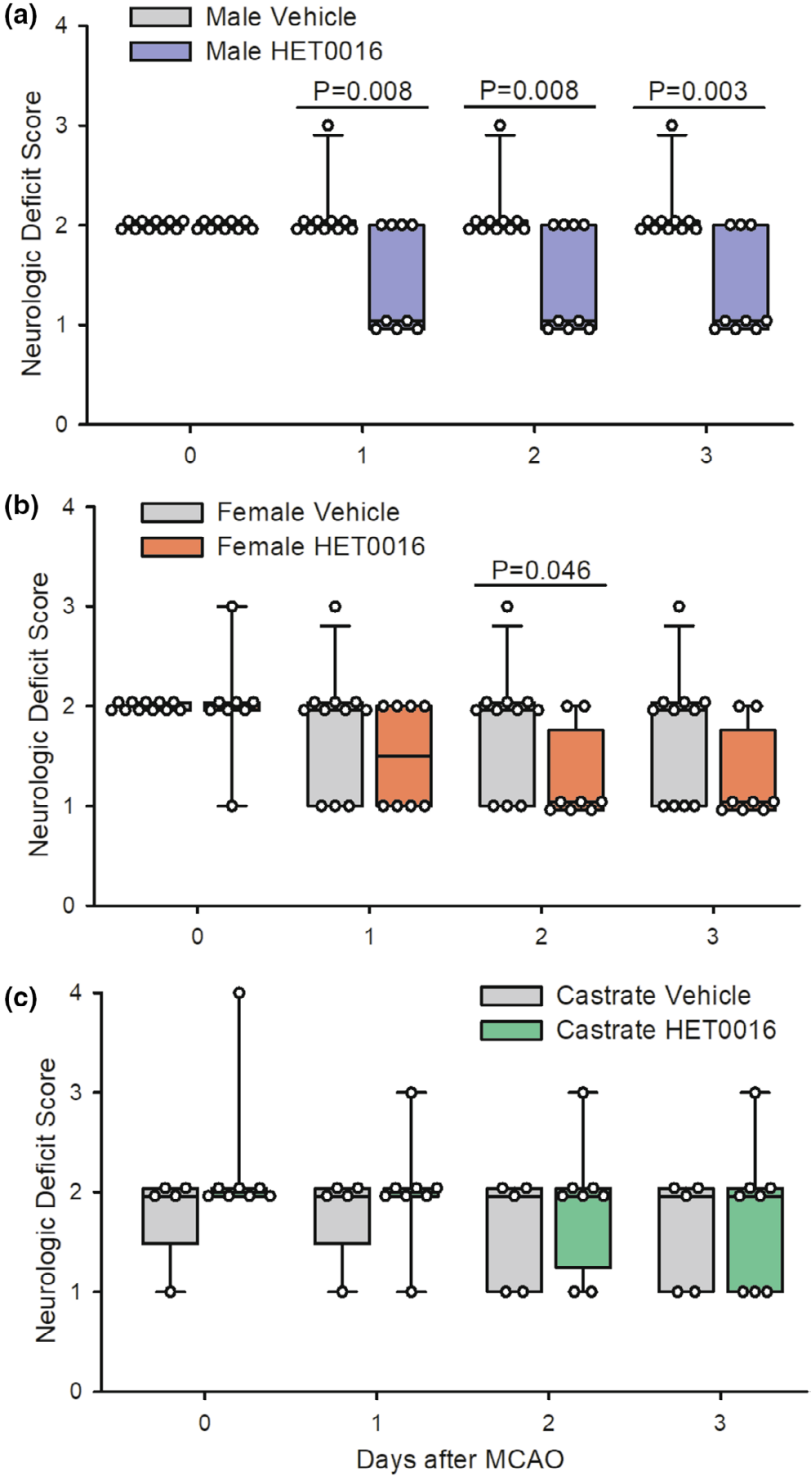
Box‐whisker plots (quartiles, whiskers 5%, 95%) and data from individual rats are shown for the neurologic deficit score on Day 0 (day of treatment) and Day 1, 2, and 3 in male rats (a), female rats (b), and castrated male rats (c) subjected to 2 h of middle cerebral artery occlusion (MCAO) and treated with either vehicle or the 20‐HETE synthesis inhibitor HET0016 (1 mg/kg intraperitoneally at 10 min prior to reperfusion and a second dose at 3 h of reperfusion). *P* values derived from the Mann–Whitney test.

The neurologic deficit score in male rats treated with HET0016 was not different from male rats treated with vehicle on day 0 (the day of the stroke; *p* = 1.0). The deficit score remained stable over the next 3 days in the vehicle‐treated males. However, compared to the vehicle group, the deficit was significantly decreased in male rats treated with HET0016 at Day 1 (*p* = 0.008), Day 2 (*p* = 0.008), and Day 3 (*p* = 0.003) after MCAO (Figure 2a). In contrast, the deficit score in HET0016‐treated female rats was not different from the vehicle‐treated females at Day 0 (*p* = 1.0), Day 1 (*p* = 0.28), or Day 3 (*p* = 0.10); at Day 2, there was a modest decrease with HET0016 treatment (*p* = 0.046; Figure 2b). In castrated males, there was no significant effect of HET0016 treatment at any day of recovery (Figure 2c).

**FIGURE 3:**
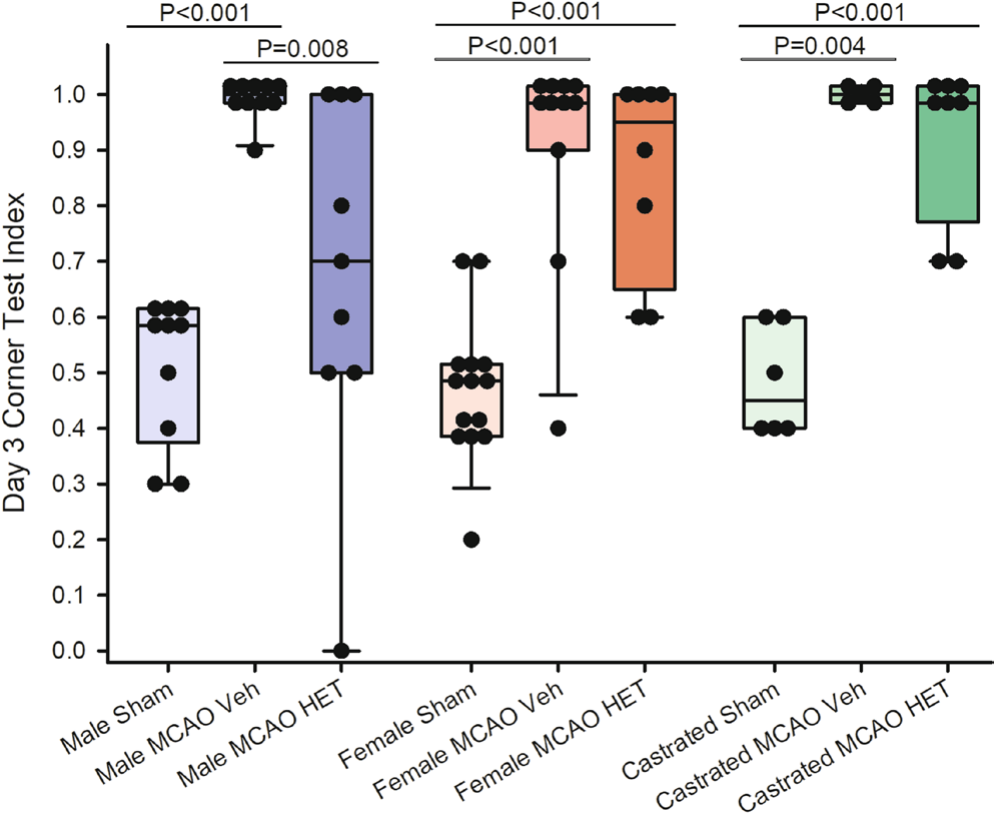
Box‐whisker plots (quartiles, whiskers 5%, 95%) and data from individual rats are shown for corner test turning preference index [right turns/(left + right turns)] 3 days after surgery in male, female, and castrated male rats undergoing sham surgery or middle cerebral artery occlusion (MCAO) with vehicle (Veh) or HET0016 (HET) treatment (1 mg/kg intraperitoneally at 10 min prior to reperfusion and a second dose at 3 h of reperfusion). *p* values derived from the Mann–Whitney test.

Before sham surgery or MCAO surgery, turning preference in the corner test was balanced in all groups, with the equipoise 0.5 fractional turning preference being included within the interquartile range (Table 2). Two‐way ANOVA was applied to the post‐surgical corner test data with sex as one factor and treatment (sham surgery, MCAO+vehicle, MCAO+HET0016) as a second factor. However, the normality and equal variance test failed. The non‐parametric Mann–Whitney test instead was used to compare the 3 treatment levels within the same sex/castrated group. At 3 days after sham surgery, turning preference remained close to 0.5 in intact and castrated male rats and female rats (Figure 3). At 3 days after MCAO, turning preference to the right (ipsilateral to the stroke) increased in all groups treated with vehicle, and the values significantly differed from the corresponding sham values. However, in males treated with HET0016 after MCAO, the asymmetric turning preference index was significantly less than that treated with vehicle (*p* = 0.008) and was not significantly different from the index in the male shams (*p* = 0.12). In females and castrated males, the turning preference index in those treated with HET0016 was not significantly different from those treated with vehicle and remained significantly increased relative to their respective sham groups.

**TABLE 2:** Median [interquartile range] of corner test turning preference index [right turns/(left + right turns)] in male, female, and castrated male rats prior to sham surgery or middle cerebral artery occlusion (MCAO) with vehicle (Veh) or HET0016 (HET) treatment at reperfusion.

	Male	Female	Castrate
Sham	0.50 [0.48–0.60]	0.50 [0.48–0.60]	0.55 [0.40–0.60]
MCAO+Vehicle	0.55 [0.48–0.60]	0.50 [0.50–0.60]	0.50 [0.40–0.55]
MCAO+HET0016	0.50 [0.30–0.55]	0.50 [0.40–0.58]	0.50 [0.43–0.58]

**FIGURE 4:**
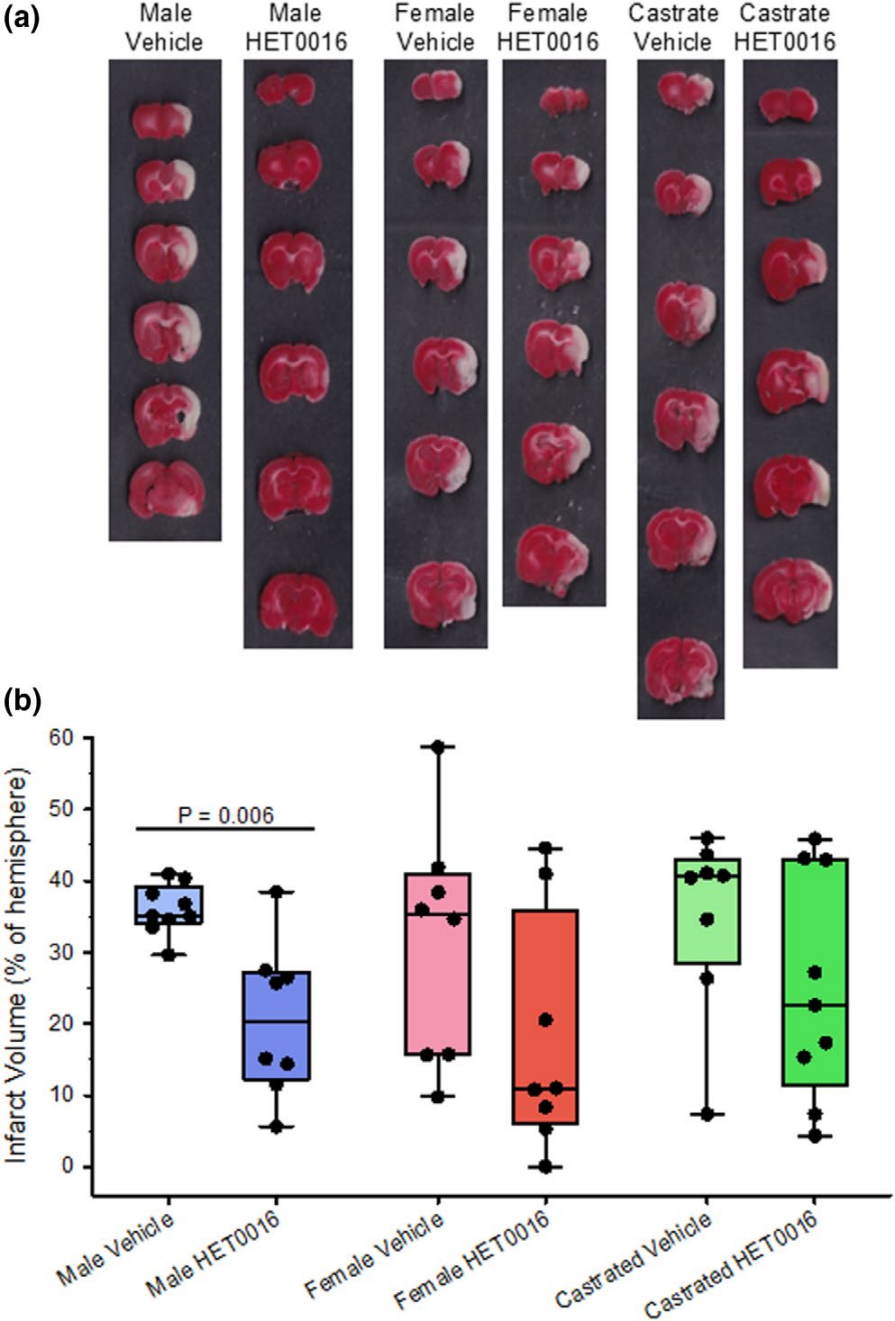
(a) Representative images of TTC‐stained serial brain slices of male, female, and castrated male rats subjected to 2 h of middle cerebral artery occlusion with 3 days of reperfusion and treated with vehicle or the 20‐HETE synthesis inhibitor HET0016 (1 mg/kg intraperitoneally at 10 min prior to reperfusion and a second dose at 3 h of reperfusion). (b) Box‐whisker plots (quartiles, whiskers 5%, 95%) and data from individual rats are shown for infarct volume. *p* values derived from the Mann–Whitney test.

Representative coronal sections of brains stained with TTC and the infarct volume data are shown in Figure 4. Two‐way ANOVA with sex/castration as one factor and vehicle/HET0016 as a second factor indicated an effect of drug treatment (*p* = 0.018) and no significant interaction with sex. However, the SD in the vehicle‐treated females and HET0016‐treated females was 16, whereas it was only 3.5 in the vehicle‐treated males and 11 in the HET0016‐treated males. This 4‐fold difference in the range of SD (16‐fold difference in variance) makes it difficult to interpret whether there is no true sex interaction. Hence, we used the Mann–Whitney test to compare infarct volume within each sex/castrated group. In intact male rats treated with HET0016, infarct volume was significantly reduced compared to those treated with vehicle (*p* = 0.006). However, HET0016 treatment had no significant effect on infarct volume in female rats (*p* = 0.16) or in castrated male rats (*p* = 0.27). Thus, the effect of HET0016 treatment was most consistent in intact males; a larger sample size would be needed to ascertain whether there is a true effect of treatment in females and castrates.

### 3.2 *Cyp4a* isoform gene expression after MCAO

**FIGURE 5:**
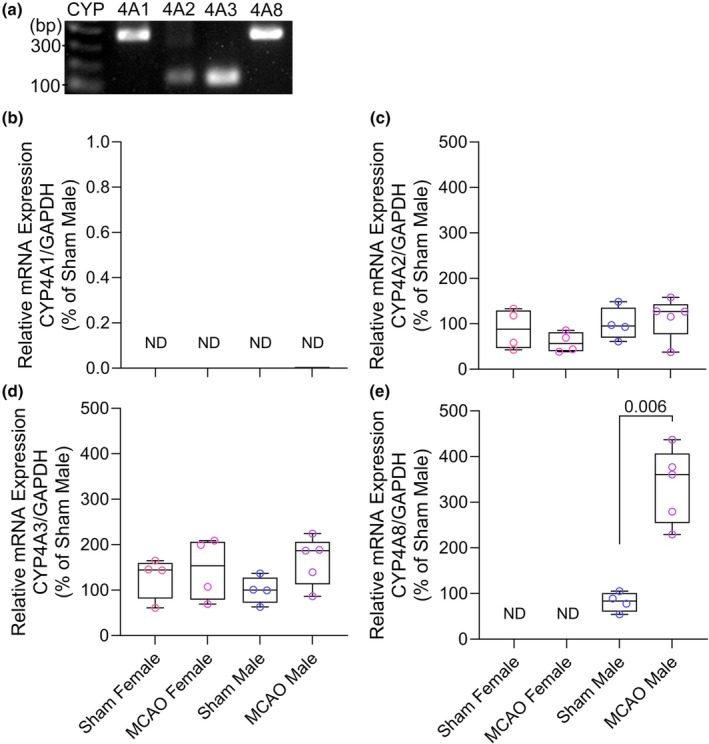
(a) The selected primers provided a positive RNA signal for each of the four *Cyp4a* genes in rat renal tissue. B–E. RT‐PCR results in brain tissue. Data points from individual rats and box‐whisker plots (quartiles, whiskers 5%, 95%) are shown for the relative expression of *Cyp4a1* (b), *Cyp4a2* (c), *Cyp4a3* (d), and *Cyp4a8* (e) after sham neck surgery in 4 female and 4 male brains and after 2 h of middle cerebral artery occlusion (MCAO) and 6 h of reperfusion in 4 female and 4 male brains. ND, not detectable. *p* value derived from the Mann–Whitney test.

To determine whether gene expression of the four rat isoforms of *Cyp4a* changes after stroke in a sex‐dependent manner, RT‐PCR was performed on brain tissues obtained after 2 h of MCAO plus 6 h of reperfusion and at an equivalent time after sham surgery. The *Cyp4a1* gene was not detectable in female or male rat brains with or without stroke (Figure 5b). The *Cyp4a2* and *Cyp4a3* genes were detectable in the female and male surgical sham brains, but there was no significant change at 6 h after reperfusion from MCAO (Figure 5c,d). In contrast, the *Cyp4a8* gene increased 3‐ to 4‐fold after reperfusion in male brains, but it was not detectable in female sham or stroked brains (Figure 5e).

**FIGURE 6:**
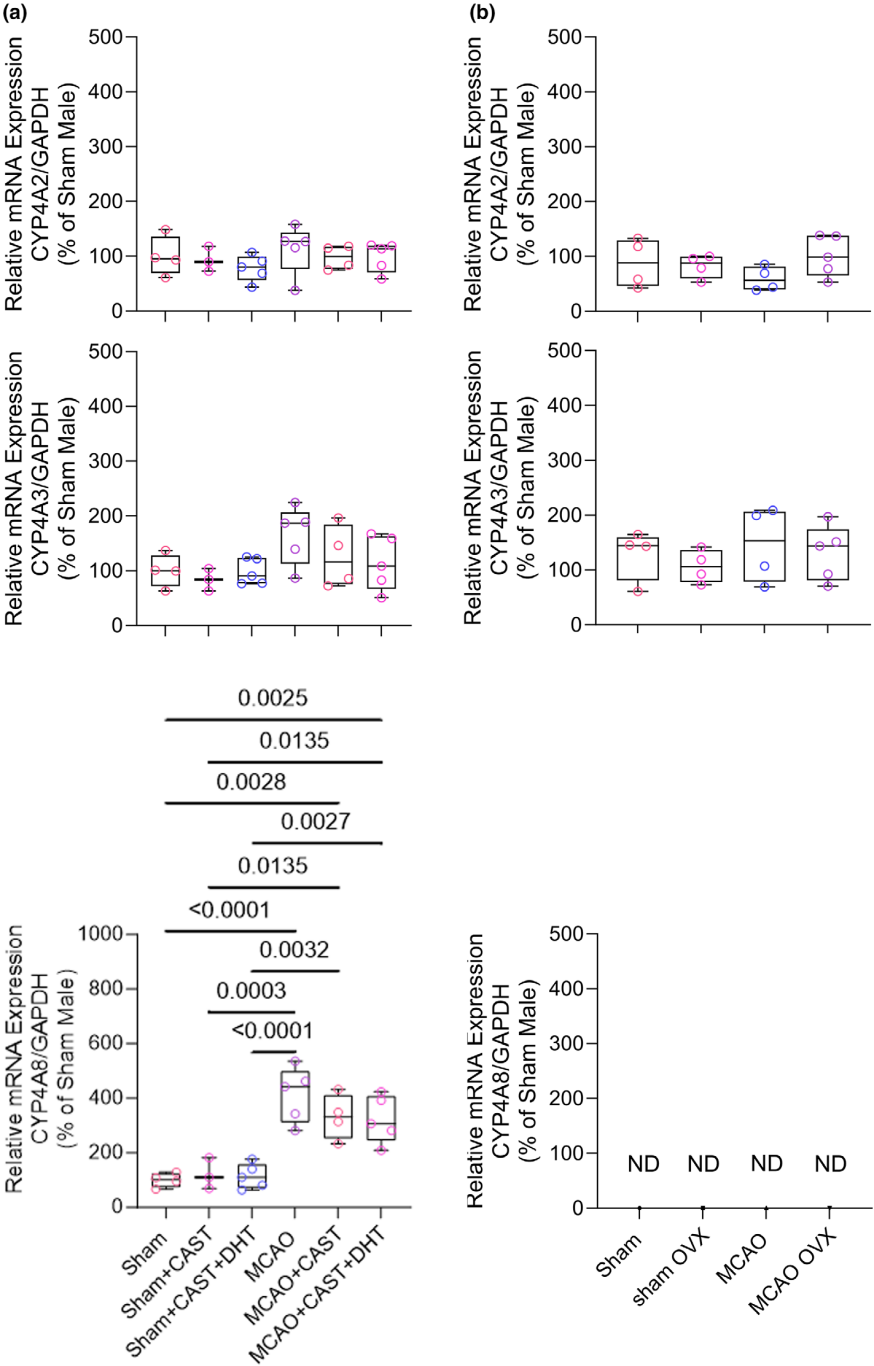
Data points from individual rats and box‐whisker plots (quartiles, whiskers 5%, 95%) are shown for the relative expression of *Cyp4a2*, *Cyp4a3*, and *Cyp4a8* in brain tissue. Measurements were made after sham neck surgery or 2 h of middle cerebral artery occlusion (MCAO) and 6 h of reperfusion. (a) Data from 4 intact males, 3 castrated (CAST) males, and 4 castrated males receiving 5α‐dihydrotestosterone (DHT) that then underwent sham surgery, and from 5 intact males, 4 CAST males, and 5 CAST males receiving DHT that then underwent MCAO. *p* values derived from the Mann–Whitney test. For the male rats, there were no significant differences among the 3 MCAO groups for the *Cyp4a2* and *Cyp4a3* genes. (b) Data from 4 intact and 4 ovariectomized (OVX) female rats that underwent sham surgery, and from 4 intact and 5 OVX female rats that underwent MCAO surgery. For the female rats, there was no significant differences between the intact and OVX groups or between the sham and MCAO groups for *Cyp4a2* and *Cyp4a3*; *Cyp4a8* was [not detectable (ND).

To determine whether the gene expression response to stroke was altered by androgens, RT‐PCR was performed on male rats after castration with or without androgen replacement with DHT. In the surgical sham groups, castration with or without DHT treatment had no significant effect on gene expression of any of the *Cyp4a* isoforms (Figure 6a). The expression of *Cyp4a2* and *Cyp4a3* genes after stroke in brains from castrated rats and castrated rats treated with DHT were not significantly different from that in the corresponding sham groups. However, *Cyp4a8* gene expression increased approximately 3‐fold after stroke in the castrated males and in the castrated males treated with DHT relative to their corresponding sham groups. This increase was not significantly different from the non‐castrated males. Thus, the expression of the *Cyp4a8* gene in brain does not appear to be influenced by androgen status.

The inconsistent effect of HET0016 on outcomes from stroke in female rats may have been due to the timing of the stroke relative to the estrous cycle. To determine whether the loss of estrogen would influence the *Cyp4a* expression after stroke, RT‐PCR was performed in ovariectomized female rats after sham surgery or MCAO. Expression of *Cyp4a2* and *Cyp4a3* failed to significantly increase after MCAO relative to the sham group, and the levels of expression were not different from that in the corresponding sham and MCAO intact female rats (Figure 6b). Expression of *Cyp4a*8 remained undetectable in brains from ovariectomized sham and MCAO rats.

### 3.3 *Cyp4a* isoform gene expression in murine cell cultures

**FIGURE 7:**
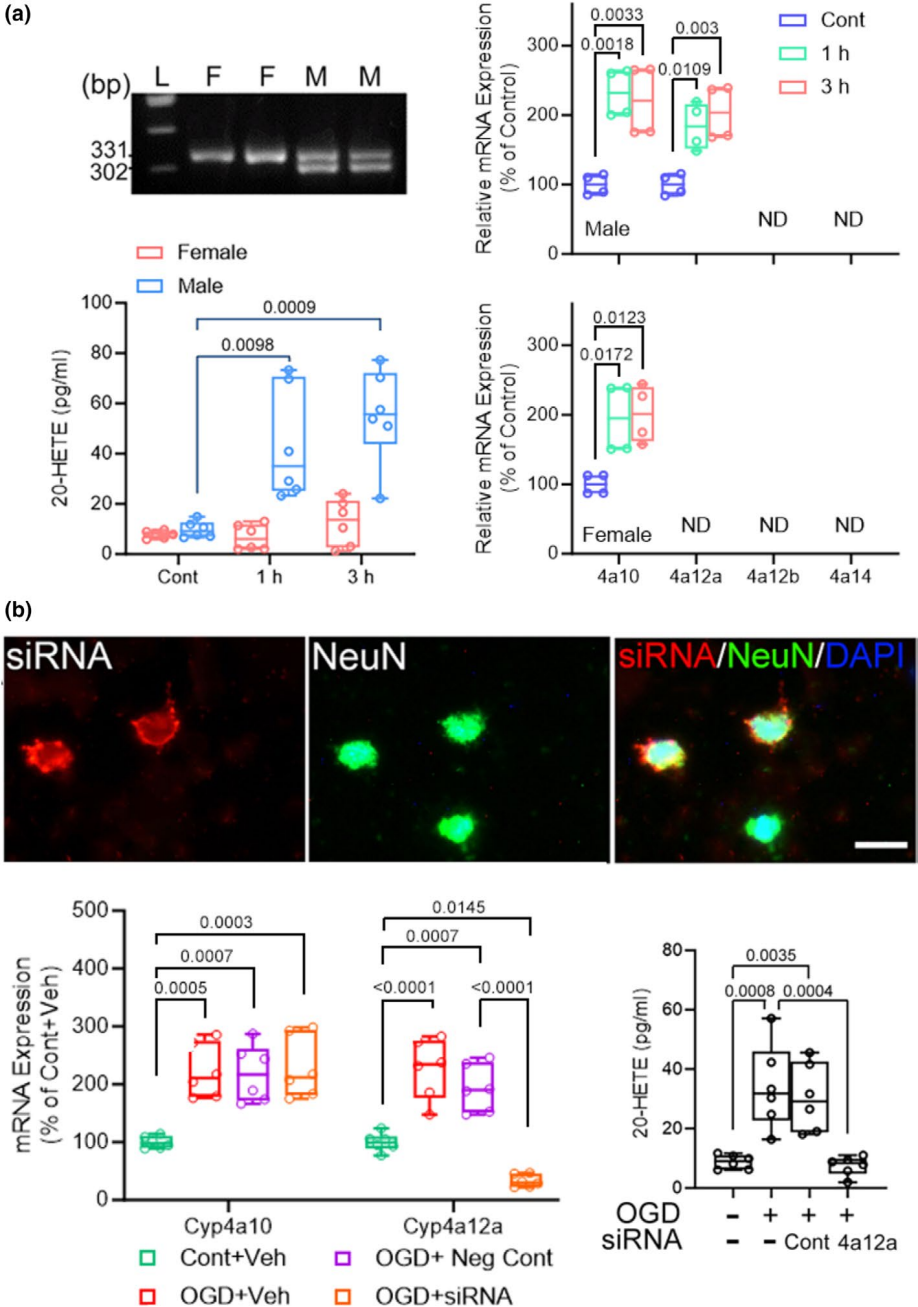
(a) Primary cortical neurons were isolated from male and female embryos and sex confirmed by PCR for Jarid1c (331 bp, X‐linked) and Jarid1d (302 bp, Y‐linked). Individual data points with box‐and‐whisker plots (quartiles, whiskers 5%, 95%) from independent cell cultures show relative mRNA levels of Cyp4a10, Cyp4a12a, Cyp4a12b, Cyp4a14 (*n* = 4) and 20‐HETE concentration (*n* = 7 control, *n* = 6 at 1 and 3 h reoxygenation) under control conditions or after 1 h of oxygen–glucose deprivation (OGD) at the indicated 1 or 3 h of reoxygenation. (b) Cy3‐labeled siRNA signals co‐localized with NeuN at 1 day after transfection. Cyp4a12a (4a12a) siRNA reduced Cyp4a12a mRNA and decreased 20‐HETE concentration in male neurons 3 h after OGD (*n* = 6 independent cell cultures per group). M, male; F, female; L, DNA ladder; ND, not detectable; Cont, control. *p* values derived from the Mann–Whitney test. Scale bar = 20 μm.

To determine the cell type in which specific Cyp4a isoforms are altered, we did not perform immunostaining for CYP4A protein because the CYP4A antibodies immunocross‐react with other isoforms. Instead, we performed OGD on primary cultures of neurons, astrocytes, and microglia to distinguish gene expression in specific cell types. In previous work with primary neuronal cultures derived from mouse embryos of mixed sex, we found selective upregulation of the *CYP4a12a* after OGD (Zhang, Falck, et al., 2017). This gene has homology with the rat *Cyp4a8* gene and human *Cyp4a11* gene (Holla et al., 2001), and it is known to ω‐hydroxylate arachidonic acid (Muller et al., 2007). Here, we extended this work by using murine neuronal cultures derived from only male pups or from only female pups. The sex of the neuronal cultures was verified by PCR amplification of X‐linked Jarid1c (331 bp) and Y‐linked Jarid1d (302 bp) genes (Figure 7a) (Clapcote & Roder, 2005). Male mouse neurons expressed *Cyp4a10* and *Cyp4a12a*, whereas female neurons expressed only *Cyp4a10* under control conditions. *Cyp4a12b* and *Cyp4a14* were undetectable in neurons of either sex. At 1 and 3 h of reoxygenation after 1 h of OGD, *Cyp4a10* and *Cyp4a12a* increased in male neurons associated with an increase in 20‐HETE. In female neurons, *Cyp4a10* expression increased but was not associated with an increase in 20‐HETE. This finding is consistent with the low capacity of *Cyp4a10* to use arachidonic acid as a substrate (Muller et al., 2007). Moreover, OGD failed to induce *Cyp4a12a* expression in female neurons or *Cyp4a12b* or *Cyp4a14* in either male or female neurons. To better define the role of CYP4A12a in increasing 20‐HETE levels after OGD in male neurons, the neurons were transfected with siRNA for *Cyp4a12a* (Figure 7b). The transfection prevented the increase in *Cyp4a12a* gene expression and blocked the increase in 20‐HETE after OGD. Thus, the OGD data in mouse neurons corroborated the in vivo rat data showing selective *Cyp4a* gene induction in male brain and further demonstrated that the induced gene in mouse neurons played a role in augmenting 20‐HETE levels.

**FIGURE 8:**
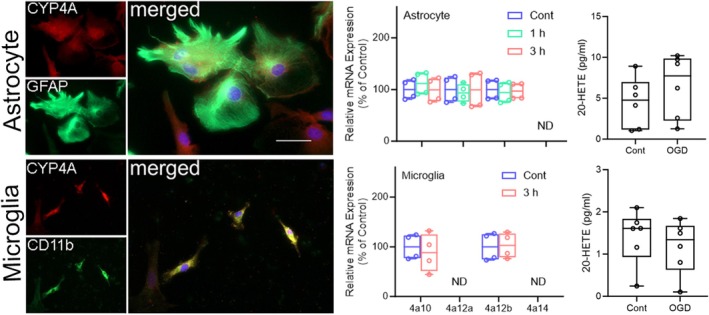
CYP4A protein colocalizes with GFAP‐stained cultured astrocytes and with CD11b‐stained cultured microglia. Individual data points with box‐and‐whisker plots (quartiles, whiskers 5%, 95%) from independent cell cultures show relative expression of Cyp4a10, Cyp4a12a, Cyp4a12b, and Cyp4a14 (*n* = 4 cultures per group) and 20‐HETE concentration (*n* = 6 cultures per group) in astrocytes and microglia under control (Cont) conditions or at 1 or 3 h of reoxygenation after 3 h oxygen–glucose deprivation (OGD). OGD data were not significantly different from corresponding Cont by Mann–Whitney test. ND, not detectable. Scale bar = 40 μm.

OGD was also performed on mixed sex cultures of mouse astrocytes and microglia. Because astrocytes and microglia are less vulnerable to OGD than neurons, the duration of OGD was extended to 3 h. Astrocytes were found to express *Cyp4a10*, *Cyp4a12a*, and *Cyp4a12b* under control conditions, but their expression did not increase after OGD with up to 3 h of reoxygenation (Figure 8). Microglia expressed *Cyp4a10* and *Cyp4a12b* under control conditions, but their expression also did not increase after OGD with up to 3 h of reoxygenation. Furthermore, the levels of 20‐HETE did not significantly increase after OGD in either astrocytes or microglia. Thus, rapid induction of *Cyp4a* isoforms and increased 20‐HETE levels after OGD were selective for neurons.

## 4 DISCUSSION

This study has several major new findings. First, MCAO and reperfusion induce *Cyp4a8* in the brain of male rats, and inhibition of CYP4A ω‐hydroxylase activity with administration of HET0016 at reperfusion reduces infarct volume and improves neurologic outcome. Second, the female rat brain does not express *Cyp4a8*; it is not induced by MCAO, and HET0016 administration at reperfusion has a less consistent, non‐significant effect on infarct volume and neurologic outcome at 3 days in female rats. Third, neither castration, with or without DHT, nor ovariectomy alters the expression of the various *Cyp4a* isoforms in sham controls or after MCAO. Fourth, OGD in cultured neurons derived from sex‐separated mouse embryos revealed selective induction of the mouse *Cyp4a* homolog *Cyp4a12a* gene in male neurons, but not in female neurons, and knocking down expression of this gene blocked the OGD‐induced increase in 20‐HETE levels. Fifth, OGD did not increase *Cyp4a* gene expression or increase 20‐HETE levels in cultured mouse astrocytes or microglia.

### 4.1 Sex‐dependent effects in stroke

Multiple mechanisms are purported to be involved in sex differences in cerebral ischemic injury (Manole et al., 2011). Among these are estrogen's vasodilatory and neuronal protective effects (Alkayed et al., 1998, 2001; Rusa et al., 1999), adverse effects of androgens in young rodents (Cheng et al., 2007, 2009), greater ROS generation and glutathione depletion in male brain (Du et al., 2004), greater expression of soluble epoxide hydrolase in young male rodents than in young female rodents (Fairbanks et al., 2012), and other genetic differences. The present study indicates that selective expression and ischemic induction of *Cyp4a8* in male brain may be another factor.

CYP4A8 protein has the capability to generate 20‐HETE by ω‐hydroxylation of arachidonic acid (Yamaguchi et al., 2002). The selective increase in the expression of the *Cyp4a8* gene evoked by MCAO compared to the other isoforms and its occurrence only in male rat brain were striking. Some similarities in sex differences Cyp4A isoforms have been found in the renal vasculature. Androgen‐induced hypertension and endothelial dysfunction are associated with *Cyp4a8* upregulation in renal arteries in rats (Nakagawa et al., 2003) and with its homolog *Cyp4a12* in mice (Holla et al., 2001). Inhibiting 20‐HETE synthesis with HET0016 reduced androgen‐induced hypertension and endothelial dysfunction. However, one difference in our study was that the MCAO‐evoked increase in *Cyp4a8* expression was still present in the brain after castration and that androgen replacement with DHT did not further increase expression. Thus, in contrast to renal artery, androgens did not appear to have a major role in the selective *Cyp4a8* expression response in male rat brain. However, one paradoxical finding was that while upregulation of *Cyp4a8* was still present in male rats after castration, HET0016 treatment no longer provided the same consistent benefit seen in intact males. This raises the possibility that other adverse signaling pathways resistant to HET0016 actions are engaged after the loss of testosterone. We did not further explore this potential adverse mechanism.

Ovariectomy has been shown to increase expression of soluble epoxide hydrolase (Koerner et al., 2008; Yang et al., 2018), which metabolizes the neuroprotective epoxide free fatty acids, such as eicosatrienoic acids. Suppression of soluble epoxide hydrolase may be one of the mechanisms by which estrogens are neuroprotective (Zhang et al., 2009). To address whether female sex hormones alter the expression of *Cyp4a* isoforms, we compared intact and ovariectomized female rats. We found no differences in expression of the *Cyp4a* isoforms. Thus, the lack of an increase in *Cyp4a8* after MCAO in female rats appears to be due to innate differences between male and female neurons rather than effects of sex hormones.

### 4.2 Sex‐dependent effects of OGD

Sex‐dependent differences in sensitivity of primary neuronal culture to OGD have been described (Fairbanks et al., 2012). Here, we show sex differences in the expression of one of the mouse *Cyp4a* genes, with *Cyp4a12a* being selectively expressed in male neurons and being induced by OGD in male neurons and not female neurons. *Cyp4a10* was expressed in both male and female neurons and was induced in both sex neurons, but CYP4A10 protein has poor capacity to use arachidonic acid as a substrate (Muller et al., 2007). The observation that 20‐HETE increases in the media of only male neurons after OGD and that knockdown of *Cyp4a12a* blocks the increase in 20‐HETE indicates that this isoform is primarily responsible for the early increase in 20‐HETE. Both CYP4A12a and CYP4A12b protein are capable of generating 20‐HETE. Although *Cyp4a12b* is expressed in cultured astrocytes and microglia, 3 h of OGD was not sufficient to induce this gene and was not sufficient to increase 20‐HETE levels in the media. Collectively, these data indicate that neurons are the main source of the early increase in 20‐HETE induced by ischemia/reoxygenation and that this source is the result of genetic differences. This conclusion derived from murine cultures is consistent with the lack of effect of castration and ovariectomy on the various *Cyp4a* isoforms in brain tissue from sham or stroked rats and implies that a sex difference in *Cyp4a* gene expression occurs across rodent species.

### 4.3 Actions of 20‐HETE

The actions of 20‐HETE in brain have primarily focused on its role in the neurovascular unit. CYP4A1 is expressed in cerebrovascular smooth muscle where 20‐HETE acts through activation of PKC and opening of L‐type Ca^2+^ channels to augment myogenic tone during increases in transmural pressure (Gebremedhin et al., 2000; Lange et al., 1997). During neuronal activation, the release of nitric oxide is thought to inhibit CYP4A and reduce myogenic tone (Alonso‐Galicia et al., 1999), thereby allowing the hyperemic response associated with neuronal activity (Liu et al., 2008). This suppression of 20‐HETE synthesis is postulated to occur not only at the level of pial and penetrating arterioles, but also at precapillary pericytes that regulate capillary red blood cell flux (Hall et al., 2014). In Sprague Dawley rats, a portion of these pericytes at capillary network entry sites express CYP4A, and those that express CYP4A have fewer red blood cells in their downstream capillaries on post‐mortem examination (Gonzalez‐Fernandez et al., 2020). Thus, it is further postulated that in the setting of ischemia, pericyte constriction sufficient to limit red blood cell flux may contribute to the no‐reflow phenomenon and heterogeneous oxygen delivery at the microcirculatory level. For example, in a study of male and female mice undergoing 1 h MCAO and 24 h reperfusion, capillary dilation in response to neuronal activation was impaired but could be restored with HET0016 or a nitric oxide donor (Li et al., 2021). However, this study was not powered to detect a possible sex difference in capillary responses nor was infarct volume or neurobehaviour reported. Moreover, the CYP4A isoform expressed in pericytes that limit capillary perfusion, at least in rats, is thought to primarily be CYP4A1 (Gonzalez‐Fernandez et al., 2020). Furthermore, inhibitors of ω‐hydoxylase activity have been found to have no effect on laser‐Doppler perfusion blood flow or pial arteriolar diameter during occlusion (Cao et al., 2009), and a benefit on blood flow after reperfusion was seen only with pretreatment of inhibitors and not with treatment started at reperfusion (Renic et al., 2009). Thus, vascular effects of HET0016 administration after reperfusion are unlikely to fully explain its beneficial effects on infarction and neurologic outcome in intact male rats, although an effect on the distribution of capillary red blood cell flux cannot be excluded as a factor contributing to the low infarct volume seen in a portion of female and castrated rats.

The benefit of HET0016 administration at reperfusion is more likely attributable to effects on neurons, where CYP4A protein expression is well documented (Gonzalez‐Fernandez et al., 2020; Renic et al., 2012; Yang et al., 2012). Although the precise physiologic role of 20‐HETE in neuronal function remains to be elucidated, 20‐HETE is known to phosphorylate the NR1 subunit of the NMDA receptor and to decrease Na,K‐ATPase activity via PKC‐mediated phosphorylation (Yang et al., 2012). Since Na,K‐ATPase inhibition would promote neuronal depolarization, it could promote excitotoxicity. Indeed, in primary neuronal cultures and hippocampal brain slices exposed to OGD, 20‐HETE contributes to the generation of reactive oxygen species and cell death (Renic et al., 2012; Zhang, Falck, et al., 2017); these effects were dependent on activation of TRPV1 receptors (Zhang et al., 2018). The 20‐HETE–dependent generation of reactive oxygen species was suppressed by an inhibitor of NADPH oxidase (NOX) (Zhang et al., 2018). Interestingly, NOX also played a role in endothelial dysfunction associated with *Cyp4a8* upregulation in renal arteries (Singh et al., 2007), suggesting common downstream signaling. Greater oxidative and nitrosative stress has been reported in male neurons compared to female neurons after exposure to excitotoxic stimuli or OGD, which leads to more rapid cell death by programmed cell necrosis (Du et al., 2004; Li et al., 2005). Thus, the upregulation of *Cyp4a8* selectively in male brains after stroke may be one of the upstream mechanisms for enhanced excitotoxic cell death reported in cultured male neurons (Du et al., 2004; Li et al., 2005) and in enhanced programmed neuronal necrosis reported after MCAO in male animals (Yuan et al., 2009).

### 4.4 Limitations of the study

Some limitations of this study should be noted. We did not measure laser‐Doppler flow, arterial blood pressure, or arterial blood gases in this study because we wanted to limit the duration of anesthesia, which can suppress arterial blood pressure and possibly collateral blood flow. Furthermore, long‐term neurobehavior recovery with more complex tasks was not performed. In addition, sex differences in stroke outcome change during aging. For example, cerebral microvessels from aged spontaneously hypertensive female rats exhibited greater 20‐HETE production capacity than their young counterparts, which had lower arterial pressure (Yanes et al., 2011). Another consideration common to most preclinical stroke studies is that attrition from mortality may bias the results if those that died had worse injury. Although the Kaplan–Meier analysis indicated no significant overall difference in mortality, the mortality was numerically greatest in the male rats treated with HET0016. Thus, we cannot exclude a bias if those that died prematurely had worse injury than the survivors.

*Cyp4a1* is known to be expressed in cerebral arteries and arterioles in Sprague Dawley rats (Gonzalez‐Fernandez et al., 2020). We did not detect *Cyp4a1*, presumably because the signal from the arterioles was too dilute in our whole tissue samples. The primer that was used did provide a signal in renal tissue samples. Moreover, the current results pertain to young, healthy rats. Whether sex differences in the role of 20‐HETE in reperfusion injury persist during aging or are altered by cardiovascular comorbidities remains to be investigated. With regard to hypertension, *Cyp4a1* and *4a8* are known to be upregulated in cerebral arteries of stroke‐prone, spontaneously hypertensive male rats, wherein augmented 20‐HETE production appears to contribute to vascular oxidative stress and vascular reperfusion injury (Dunn et al., 2008). Whether the vascular effects of 20‐HETE during ischemia may be more prominent in renal models of hypertension remains to be studied.

It should also be noted that in addition to isoforms of CYP4A, some isoforms in the CYP4F family are capable of generating 20‐HETE (Cheng et al., 2014) and have been studied mostly in kidney and liver (Kalsotra et al., 2005; Stec et al., 2003). Expression of *Cyp4f4* and *Cyp4f5* is decreased in liver in a model of systemic inflammation and is decreased in the hippocampus in the controlled cortical impact model of brain injury (Cui et al., 2003). We did not investigate whether CYP4F isoforms are changed in the brain by ischemic stroke.

This study was designed to be exploratory in nature for assessing whether the role of 20‐HETE in injury from stroke is sex‐dependent. While inhibition of 20‐HETE synthesis reduces ROS and cell death in cultured neurons and brain slices after OGD, possible sex‐dependent effects of HET0016 on ROS generation, the various pathways of programmed cell death, blood–brain barrier injury, and inflammation remain to be investigated in the setting of ischemic stroke in vivo.

## 5 CONCLUSION

The results of this study demonstrate that one of the four *Cyp4a* genes, *Cyp4a8*, is selectively expressed and upregulated after transient ischemic stroke in the brain of male rats but not in the brain of female rats. Selective sex‐dependent gene upregulation was corroborated by OGD data in cultured male neurons. Furthermore, administration of the 20‐HETE synthesis inhibitor HET0016 reduced infarct volume and improved functional outcome on both the neurologic deficit score and corner test at 3 days of recovery more consistently in male rats. 20‐HETE has also been implicated in the pathogenesis of ferroptosis after intracerebral hemorrhage (Han et al., 2021), neuroinflammation after traumatic brain injury (Shu et al., 2019), and cerebral vasospasm after subarachnoid hemorrhage (Takeuchi et al., 2005). It will be important to more closely evaluate potential sex differences attributable to 20‐HETE in these other models of acute brain injury.

## AUTHOR CONTRIBUTIONS

Rongrong Zhang acquired data, analyzed data, and edited and approved the manuscript. Yanrong Shi acquired data and edited and approved the manuscript. Suyi Cao acquired data and approved the manuscript. Raymond C. Koehler conceptualized the project, analyzed the data, created figures, and drafted and approved the manuscript. Zeng‐jin Yang conceptualized the project, obtained funding, acquired data, analyzed data, created figures, and drafted and approved the manuscript.

## FUNDING INFORMATION

This work was supported by internal funding from a Stimulating and Advancing ACCM Research (StAAR) grant from the Department of Anesthesiology and Critical Care Medicine at Johns Hopkins University. Rongrong Zhang received support from The First Affiliated Hospital of Chongqing Medical University, Chongqing, China.

## CONFLICT OF INTEREST

The authors declare no conflicts of interest.

## Data Availability

Data are available from the corresponding author.
